# COVID-19 severity in patients with chronic lymphocytic leukemia treated with venetoclax: a single-center observational cohort study

**DOI:** 10.1007/s00277-024-05738-4

**Published:** 2024-04-18

**Authors:** Sophie Thau, Christian Bjørn Poulsen, Christian Brieghel, Morten Kranker Larsen, Lothar Wiese, Xiaohui Chen Nielsen, Lars Møller Pedersen

**Affiliations:** 1 Department of Hematology, Zealand University Hospital, Roskilde, Denmark; 2 Department of Infectious Diseases, Zealand University Hospital, Roskilde, Denmark; 3 Department of Clinical Microbiology, Zealand University Hospital, Slagelse, Denmark; 4 Department of Clinical Medicine, University of Copenhagen, Copenhagen, Denmark

**Keywords:** CLL (chronic lymphocytic leukemia), Small lymphocytic lymphoma, Venetoclax, COVID-19

## Abstract

Patients with chronic lymphocytic leukemia (CLL) are at high risk of developing severe COVID-19. The present study was undertaken to elucidate COVID-19 related morbidity and mortality in CLL patients treated with venetoclax. We present a single-center study of 108 patients with small lymphocytic lymphoma or CLL treated with venetoclax. Primary outcome was 30-day COVID-19 mortality. Secondary outcomes included COVID-19 severity and hospitalization rate. Forty-eight (44%) patients had PCR-verified SARS-COV-2 between March 2020 and January 2023. Thirty-six patients (75%) presented with asymptomatic/mild COVID-19 and 12 (25%) with severe/critical disease. The hospitalization rate was 46% with a 30-day mortality rate of only 4% and severe comorbidities as the primary cause of death. COVID-19 severity and mortality were similar before and during the Omicron era. High CIRS-scores (*P* < 0.02) and thrombocytopenia (*P* < 0.01) were more frequent in patients with severe/critical disease. In real-world data, most venetoclax treated patients presented with mild COVID-19. Hospitalization and mortality rates were low compared to data of general CLL populations. Our data indicate that venetoclax was a safe treatment option for CLL patients during the pandemic.

## Introduction

Chronic lymphocytic leukemia (CLL) patients diagnosed with severe acute respiratory syndrome coronavirus-2 (SARS-CoV-2) are at high risk of hospitalization and death [1]. Risk factors for severe coronavirus disease 2019 (COVID-19) infections including hypogammaglobulinemia, B and T-cell defects, CD4 + lymphopenia, neutropenia, innate immune dysfunction, treatment with anti-CD20 targeting agents, and age-related medical conditions are often present in CLL patients [2]. Early studies of COVID-19 and CLL have indicated high mortality rates at approximately 30% among hospitalized patients [1, 3]. The majority of COVID-19 positive CLL patients required hospitalization, especially in the beginning of the pandemic [3–5]. The rates for intensive care unit (ICU) admission and intensive treatments with oxygen supplements and mechanical ventilation were also excessive compared to patients without a hematological disease [6]. High age, poor performance status, anemia and thrombocytopenia were among risk factors for developing severe COVID-19 [7].

Over the past decade, treatment of CLL has shifted from chemoimmunotherapy to targeted therapy [8]. Targeting B cell lymphoma 2 (BCL-2) with venetoclax in combination with anti-CD20 antibodies has improved clinical outcomes in patients with CLL both in treatment naïve patients and in the relapsed/refractory setting [9, 10]. A fixed-duration venetoclax in combination with obinutuzumab is a recommended first-line treatment for patients with CLL and small lymphocytic lymphoma (SLL)​, and venetoclax in combination with rituximab is a recommended second-line treatment [9–11]. Venetoclax has demonstrated an overall acceptable toxicity profile in patients with CLL. However, among the most common grade 3/4 adverse events are neutropenia, febrile neutropenia, pneumonia, and sepsis [12]. There are few available data on COVID-19 in patients receiving venetoclax-based treatments. A study has suggested that venetoclax blocks the interaction between the SARS-CoV-2 spike (S) glycoprotein and the angiotensin-converting enzyme 2 receptor through spike protein degradation [13]. Venetoclax may therefore exhibit protective mechanisms against COVID-19 by inhibiting viral nucleic acid sequences to enter the host cell. In agreement with these observations, a recent study reported only two (7%) hospitalizations and/or deaths after contracting COVID-19 among 27 CLL patients receiving venetoclax, and no events were recorded in patients receiving nirmatrelvir plus ritonavir [14]. Other studies have proposed that treatment with venetoclax is correlated with higher immunogenicity and seroconversion rates after COVID-19 booster vaccines compared to ibrutinib [15].

Here we present real-world data of the frequency, severity and mortality of COVID-19 in an observational single-center study of CLL and SLL patients consecutively treated with venetoclax during the COVID-19 pandemic. Our data were collected during both the pre- and post-vaccine era as well as the pre-Omicron and Omicron wave.

## Methods

### Patients

Patients diagnosed with CLL or SLL and treated with venetoclax were included in an observational single-institution study. Patients were identified in the electronic patient health record platform (EPIC). Venetoclax treatment was initiated between April 2017 and December 2022. Patients were treated with standard ramp up and steady state dose of venetoclax. Dose level and duration of treatment varied depending on tolerability and efficacy.

### SARS-CoV-2 testing

COVID-19 was defined as a positive SARS-CoV-2 reverse transcription polymerase chain reaction (RT-PCR) test. Local guidelines at our institution included testing of all hematological patients upon admission or out-patient visits, and mass screening twice a week during surges. A high frequency of positive SARS-CoV-2 samples were whole genome sequenced and the information on SARS-CoV-2 including sublineage was retrieved from the Danish national microbiology database (https://www.covid19genomics.dk/nextstrain) [16]. We used a cut-off point corresponding to January 2022 to compare patients with COVID-19 before and during the Omicron era. The Omicron subvariants were dominating in Denmark since January 2022, including the most frequent variant B.1.1.529​ [4]. Prior to this, Delta B.1.617.2 (original Delta variant) and Delta AY.4.2 (Delta plus variant) were the dominating variants. The Danish COVID-19 Genome Consortium (DCGC) provided data on the SARS-CoV-2 subvariants.

### Clinical data collection

Data were collected from electronic patient records between August 2022 and January 2023. Baseline characteristics and clinical outcomes were recorded including severity and mortality of COVID-19 infections. The primary outcome of the study was 30-day COVID-19 mortality rate, defined by death within 30 days from positive PCR test. Secondary outcomes included COVID-19 severity, hospitalization rate, admission rate to ICU, 90-day COVID-19 mortality, and treatments of COVID-19. Secondary infections were recorded and defined as clinically (e.g., clinical examination, laboratory test results or imaging) or microbiologically proven infections occurring within a 2-month period after a positive PCR test.

### Statistics

All data were registered in a local secured research database (REDCap). Statistical analyses were performed in R software (version 4.2.3). Clinical variables were summarized using descriptive statistics. Categorical variables are presented as numbers and percentages and continuous variables as median and ranges or interquartile ranges (IQR). Categorical variables were analyzed by Fisher’s exact test. 30-day COVID-19 mortality rate was estimated as the proportion alive 30 days after verified SARS-CoV-2 infection. The hospitalization rate was measured as the proportion of patients with hospitalization of minimum 24-hours duration within 30-days from a positive PCR test. ICU admission rate was calculated as the proportion of patients admitted to an ICU within one month of verified infection. Time-to-event analyses were performed using Kaplan-Meier plots and log-rank test was used for the comparison of survival in different groups. The level of statistical significance was defined as *P* < 0.05.

## Results

### Patient characteristics

**Fig. 1:**
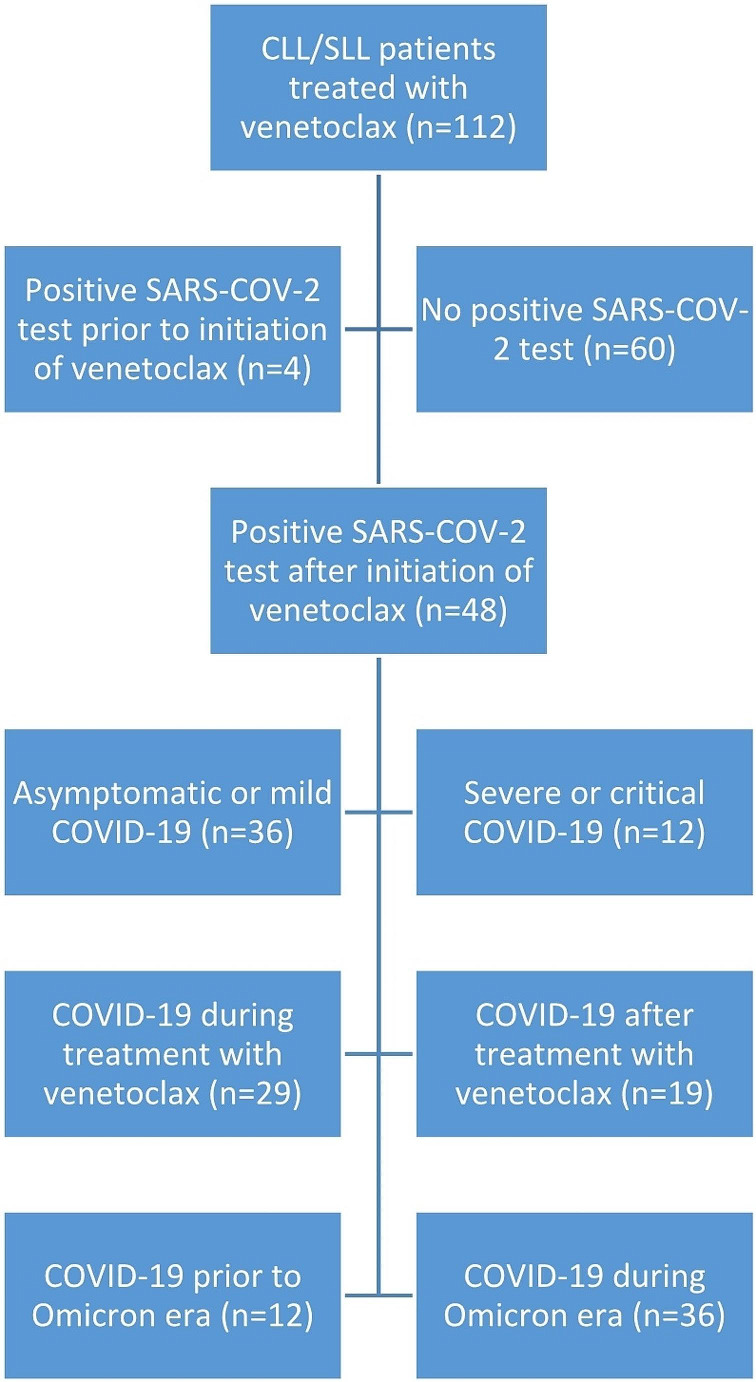
Flow chart with stratification of the study cohort

We identified 112 patients with CLL or SLL treated with venetoclax. Four patients with COVID-19 infection prior to initiation of venetoclax were excluded. Of the remaining 108 patients, 48 (44%) patients had a positive SARS-CoV-2 PCR test after initiation of venetoclax treatment. Flowchart of clinical subgroups in the study cohort is provided in Fig. 1. We identified the SARS-CoV-2 variant in 27/48 (56%) patients, with 6 cases of B.1.617.2 (Delta-variant) and 21 cases of B.1.1.529 (Omicron-variant). The distribution of SARS-CoV-2 variants was in accordance with our cut-off point with a case of B.1.617.2 (Delta-variant) detected in 2022 as the only exception. Most patients (98%) had received the recommended number of vaccines in Denmark with 92% being vaccinated with ≥ 4 doses. Sufficient vaccine antibody response measured with enzyme-linked immunosorbent assay (ELISA) was observed in 15/32 (47%).

Baseline clinical characteristics of the total cohort and for patients with and without COVID-19 are shown in Table 1. In the total cohort (*n* = 108), median (range) age was 71 years (47–89) and 77 (71%) were males. No significant differences in frequencies of baseline features were found between patients with and without COVID-19 with only a trend toward lower steady state dose of venetoclax in the cohort with COVID-19 (*P* = 0.05). The most frequent causes of dose reduction were toxicity including gastrointestinal symptoms and neutropenia. Median (range) lines of therapy prior to venetoclax were 1 (0–6). The treatment regimens were combined venetoclax and CD20-antibodies including rituximab or obinutuzumab (*n* = 95), venetoclax and BTKi including ibrutinib or acalabrutinib (*n* = 11), or venetoclax monotherapy (*n* = 9). Four patients received a rituximab-obinutuzumab-venetoclax combination, two patients received an obinutuzumab-ibrutinib-venetoclax combination, and one patient received an obinutuzumab-acalabrutinib-venetoclax combination. The median (IQR) duration of venetoclax treatment was 13 months (12–24). Median (IQR) time between initiation of venetoclax and a positive SARS-CoV-2 PCR test was 19 months (8–26).

COVID-19 infection was detected during ongoing venetoclax treatment in 29/48 (60%) and after discontinuation of venetoclax in 19/48 (40%). In 19/29 (66%) patients diagnosed with COVID-19 infection during venetoclax treatment, venetoclax was continued without changing the current dose level. In four (14%) patients, the decision was made to temporarily discontinue venetoclax treatment for a duration of 2 to 60 days. In another four (14%) patients, venetoclax was permanently discontinued when a sufficient response to treatment had already been achieved and termination of venetoclax was expected in a short period of time regardless of the appearance of the infection. The two remaining patients died shortly after discontinuation of venetoclax from causes regarded as non-related to COVID-19 infection. The hospitalization rate among patients with ongoing venetoclax treatment at the time of COVID-19 diagnosis was 48%.

**Table 1:** Clinical characteristics at initiation of venetoclax treatment

Variables	Cohort with COVID-19 (*n* = 48)	Cohort without COVID-19 (*n* = 60)	Total cohort (*n* = 108)
Age ≥70 years	26 (54%)	38 (63%)	64 (59%)
Gender male	31 (65%)	46 (77%)	77 (71%)
Binet stage C	21 (44%)	32 (53%)	53 (49%)
Genetics:			
Del(11q)	13 (27%)	17 (28%)	30 (28%)
* TP53* disruption	8 (26%)	18 (42%)	26 (35%)
Missing			34 (31%)
IGHV unmutated	26 (66%)	37 (74%)	63 (71%)
Missing			19 (18%)
CLL treatments:			
≥ 1 prior lines of therapy	28 (58%)	42 (70%)	70 (65%)
Treated with venetoclax + CD20 antibody	41 (85%)	50 (83%)	91 (84%)
Treated with venetoclax + BTK inhibitor	7 (15%)	4 (7%)	11 (10%)
Treated with venetoclax monotherapy	2 (4%)	7 (12%)	9 (8%)
Reduced venetoclax steady state (< 400 mg)	17 (35%)	13 (22%)	30 (28%)
Blood analyses:			
Hemoglobin ≤ 10 g/dL	7 (15%)	10 (17%)	17 (16%)
Missing			2 (2%)
Thrombocytes ≤ 100 × 10^9^/L	8 (17%)	11 (18%)	19 (18%)
Missing			2 (2%)
IgG ≤ 6 g/L	24 (57%)	26 (45%)	50 (50%)
Missing			8 (7%)
Supportive G-CSF	21 (44%)	25 (42%)	46 (43%)
Comorbidity:			
CIRS score ≥ 6	24 (50%)	36 (60%)	60 (56%)
History of diabetes	5 (10%)	11 (19%)	16 (15%)
History of pulmonary disease	13 (27%)	17 (28%)	30 (28%)
Active smoker	9 (19%)	9 (15%)	18 (17%)
History of cardiovascular disease	18 (38%)	26 (43%)	44 (41%)
History of kidney disease	5 (10%)	10 (17%)	15 (14%)
BMI ≥ 25	33 (69%)	38 (63%)	71 (66%)

### COVID-19 severity and mortality

Most patients presented with mild symptoms or asymptomatic COVID-19 disease (75%) and the hospitalization rate at 30 days was 46%. No significant differences in severity were detected between patients with COVID-19 during and after the pre-Omicron era (Table 2). Three patients (6%) experienced reactivation of the same SARS-CoV-2 origin within 2 months after a negative PCR test. Two patients experienced reactivation with the B.1.1.539-variant (Omicron type) and one patient had reactivation with the B.1.617-variant (Delta type) with all 3 cases of reactivation being symptomatic. Distribution of clinical characteristics at initiation of venetoclax between patients with asymptomatic/mild disease and patients with severe/critical disease is shown in Table 3. Only thrombocytopenia and high CIRS-score were found with a significantly higher frequency in patients with severe/critical disease. Treatments of the COVID-19 infection are shown in Table 4. The antiviral treatment was given according to Danish national guidelines. Patients with ongoing venetoclax treatment more frequently received treatments with preemptive anti-SARS-Cov-2 monoclonal antibodies (24/29 (83%) and 10/19 (53%), respectively; *P* = 0.04), other antiviral drugs (13/29 (45%) and 2/19 (11%), respectively; *P* = 0.02), and antibiotics (15/29 (52%) and 6/19 (32%), respectively; *P* < 0.01).

**Table 2:** COVID-19 outcomes according to SARS-CoV-2 variants

Outcome	COVID-19 pre-Omicron (*n* = 12)	COVID-19 Omicron-era (*n* = 36)	COVID-19 total cohort (*n* = 48)
Asymptomatic or mild COVID-19 disease	9 (75%)	27 (75%)	36 (75%)
Severe or critical COVID-19 disease	3 (25%)	9 (25%)	12 (25%)
Hospitalization	6 (50%)	16 (44%)	22 (46%)
Pulmonary infiltrates on X-ray	5 (42%)	8 (22%)	13 (27%)
SpO2 < 90%	1 (8%)	7 (19%)	8 (17%)
ICU admission	0	0	0

**Table 3:** Distribution of baseline clinical features at initiation of venetoclax in patients with asymptomatic/mild and severe/critical COVID-19

Variables	Asymptomatic/mild disease (*n* = 36)	Severe/critical disease (*n* = 12)	P value
Gender male	22 (61%)	9 (75%)	0.50
Binet stage C	13 (36%)	8 (67%)	0.09
*TP53* disruption	6 (17%)	1 (8%)	0.66
Missing	13 (36%)	4 (33%)	
IGHV unmutated	19 (53%)	7 (58%)	1
Missing	5 (14%)	4 (33%)	
Hemoglobin ≤ 10 g/dL	6 (17%)	1 (8%)	1
Missing	1 (3%)	1 (8%)	
Thrombocytes ≤ 100 × 10^9^/L	6 (17%)	4 (33%)	< 0.01
Missing	1 (3%)	1 (8%)	
IgG ≤ 6 g/L	22 (69%)	7 (70%)	1
Missing	4 (11%)	2 (17%)	
CIRS-score ≥ 6	14 (39%)	10 (83%)	< 0.02
Diabetes	4 (11%)	1 (8%)	1
Pulmonary disease	7 (19%)	6 (50%)	0.08
Cardiovascular disease	13 (36%)	5 (42%)	0.74
BMI ≥ 25	25 (69%)	8 (66%)	1

**Table 4:** COVID-19 treatments

Treatment	COVID-19 cohort (*n* = 48)
Dexamethasone	10 (21%)
Anticoagulants	11 (23%)
Antiviral antibodies (sotrovimab, tixagevimab/cilgavimab, casirivimab/imdevimab)	34 (71%)
Other antivirals (remdesivir, nirmatrelvir/ritonavir)	15 (31%)
Treated with oxygen supplements	10 (21%)
Antibiotics	21 (44%)
No treatments	12 (25%)

Only 2/48 (4%) patients died within 30 days of a positive SARS-CoV-2 PCR test. Both deaths were regarded as non-related to COVID-19 infection. In one case the primary cause of death was given as pulmonary cancer and in the other case as Richter’s transformation. No additional deaths were observed 90 days after a positive SARS-CoV-2 PCR test.

### Secondary infections

In the cohort of patients treated with venetoclax (*n* = 48), secondary infections were observed in 17 (35%) patients while 21 (44%) of the patients were treated with antibiotics. The infections were either verified clinically (e.g., clinical examination or pulmonary infiltrates on imaging) or by microbiological detection of pathogens. Most cases were pneumonias, upper respiratory or urinary tract infections. A pseudomembranous colitis was observed in one case. No systemic mycoses or other severe fungal infections were detected. The most common pathogen was *Haemophilus influenzae* (3 cases). All cases of secondary infections including microbiological pathogens are detailed in Table 5.

**Table 5:** Secondary infections after COVID-19 (within 2 months from positive PCR test)

Focus of infection	Detected pathogen	Clinical signs of infection
Pneumonia	*Influenza A virus*	Pulmonary infiltrates (X-ray), symptoms of pneumonia
Pneumonia	None	Pulmonary infiltrates (X-ray), symptoms of pneumonia, increasing CRP levels, decreasing SpO2-levels
Pneumonia	None	Pulmonary infiltrates (X-ray), symptoms of pneumonia, increasing CRP levels, decreasing SpO2-levels
Pneumonia	None	Pulmonary infiltrates (X-ray), symptoms of pneumonia, increasing CRP levels, decreasing SpO2-levels
Pneumonia	None	Symptoms of pneumonia, increasing CRP levels
Upper respiratory infection / otitis media	*Haemophilus influenzae*	Symptoms of upper respiratory infections and otitis media, increasing CRP levels
Urinary tract infection	*Escherichia coli*	Symptoms of urinary tract infection
Pneumonia	*Haemophilus influenzae*	Pulmonary infiltrates (X-ray), symptoms of pneumonia, increasing CRP levels, decreasing SpO2-levels
Gastroenteritis	*Rotavirus*	Symptoms of gastroenteritis, increasing CRP levels
Pneumonia	None	Pulmonary infiltrates (X-ray), symptoms of pneumonia, increasing CRP levels
Pneumonia	*Haemophilus influenzae*	Pulmonary infiltrates (X-ray), symptoms of pneumonia, increasing CRP levels, decreasing SpO2-levels
Urinary tract infection	*Citrobacter freundii, Morganella morganii*	Symptoms of urinary tract infection, increasing CRP levels
Pneumonia	*Respiratory syncytial virus*	Pulmonary infiltrates (X-ray), symptoms of pneumonia, increasing CRP levels, decreasing SpO2-levels
Pneumonia	*Streptococcus pneumoniae, Moraxella catarrhalis*	Symptoms of pneumonia, increasing CRP
Urinary tract infection and pseudomembranous colitis	*Proteus mirabilis (urine), Clostridium difficile (faeces)*	Symptoms of urinary tract infection and pseudomembranous colitis, increasing CRP
Pneumonia	None	Symptoms of pneumonia, increasing CRP
Pneumonia	None	Pulmonary infiltrates (X-ray), symptoms of pneumonia, increasing CRP levels, decreasing SpO2-levels

## Discussion

The extensive Danish testing strategy and the access to the nationwide collection of PCR data made it possible to present COVID-19 data among CLL patients treated with venetoclax. The highest rate of positive PCR tests in Denmark was 33.99% in February 2022 and the average positive rate during the entire pandemic was estimated to be 5.03% (https://ourworldindata.org/coronavirus/country/denmark).​ The testing frequency was particularly high among patients who were at high risk for developing severe COVID-19 illness, including patients with hematologic malignancies. We present real-world data from a single-institution observational study of COVID-19 infections in an unselected CLL/SLL cohort treated with venetoclax. The clinical presentation in our cohort is comparable to similar studies, except for a relatively high rate of patients with unmutated IGHV and *TP53* disruption. Almost all patients (98%) had received a full COVID-19 vaccination program. Among patients with available assessments of vaccine antibody responses, an adequate response could be demonstrated in 47%. This is consistent with a recent study reporting a serological response in > 40% of patients treated with venetoclax but humoral responses to vaccines were significantly higher in treatment-naïve CLL patients [17]. The incidence of COVID-19 in our cohort was 44%, in accordance with the general Danish population between March 2020 and March 2023 (54%) (https://www.sst.dk/da/corona/Status-og-materiale/Coronatal). Most patients presented with COVID-19 during the Omicron era (75%) and 25% of the patients developed severe/critical COVID-19 disease. Dose reductions of venetoclax were slightly more frequent in the group of patients with COVID-19 infection. The distributions of baseline clinical features were similar in patients with and without COVID-19 infection. Half of the COVID-19 patients were hospitalized but no ICU admissions were registered. Other studies of CLL patients and COVID-19 show similar hospitalization rates but notable higher ICU admission rates at 19–22% [6, 7]. Routine PCR testing at our center could have made early detection and intervention with antiviral antibodies more likely. This may have contributed to the low ICU admission rate.

The 30-day mortality rate was found to be lower than expected with only two deaths (4%), both occurring in patients with severe comorbidities as the primary cause of death. Higher mortality rates of 19–33% among CLL patients have been reported in other studies [3, 18]. In a recent large clinical trial of first-line venetoclax combinations, COVID-19 was detected in a small subgroup with 4/45 (9%) deaths in venetoclax-treated patients with COVID-19 and 2/10 (20%) deaths in chemoimmunotherapy-treated patients [11]. These mortality rates are higher compared to our data, but the study was primarily conducted during the start of the pandemic. In a recent Danish nationwide study, a CLL cohort with a positive SARS-CoV-2 PCR test and a subgroup of CLL patients tested positive at hospital test sites presented with a 30-day mortality rate of 2% and 23%, respectively [4]. The 30-day mortality of the total cohort was 4.7%, which is comparable to the findings of our study. The low mortality rates may partly be explained by most patients in both studies being diagnosed with SARS-CoV-2 in the late period of the pandemic. In this period, better treatment opportunities with antiviral antibodies were available and the milder Omicron BA.1 variant was dominant. The hospitalization rate in the Danish population-based study was slightly higher (68%) compared to our data (46%) [4]. The rates of ICU admission were 8.5% and 0%, respectively despite our cohort being characterized by patients with more severe CLL according to Binet stage, unmutated IGHV and *TP53* disruption status. This comparison, therefore, points towards a generally mild course of COVID-19 infections among patients treated with venetoclax.

In our study, patients in ongoing venetoclax treatment required more intensive treatment with antiviral antibodies, antiviral drugs and antibiotics compared to patients with COVID-19 after discontinuation of venetoclax. In general, infections are relatively common in patients treated with venetoclax. Multiple studies had shown grade 3–4 infection rates at estimated 20% in patients during treatment, most frequently pneumonia, sepsis or febrile neutropenia [9, 19]. Furthermore, cytopenia including neutropenia are one of the most common grade 3–4 adverse events related to venetoclax [20]. However, our real-life data has shown that infections with COVID-19 generally has a mild and manageable course in venetoclax treated patients and a very low mortality mostly related to accompanying comorbidities.

In vitro studies investigated the ability of venetoclax to bind and degrade the spike protein in SARS-CoV-2 [14, 21]. The pharmacological properties and the abilities to interact with the spike protein of SARS-CoV-2 were investigated. Venetoclax was able to degrade the expression of the spike protein and blocking the virus’ interaction with the ACE-2 receptor. These findings indicate that blocking the interaction between the spike protein and the ACE-2 receptor potentially prevent the virus’ entry into the host cell. Venetoclax may therefore express some properties with a protective effect against severe COVID-19 disease.

The present study has some limitations, including a small sample size, missing data on some SARS-COV-2 variants and partly missing data on antibody levels. The size of the cohort did not allow more extended analyses with most data being descriptively presented. However, our data clearly suggest that CLL patients who are treated with venetoclax generally present with manageable COVID-19 and a low mortality rate. Compared with data of general CLL populations, venetoclax-treated patients in our cohort had a mild course of COVID-19 and suggest that venetoclax is a safe treatment option for CLL patients with adequately treated COVID-19.

## Data Availability

Data were collected and managed using the REDCap electronic data capture tool hosted at Ascension. Data are available from the corresponding author upon reasonable request. The individual level data collected during the study are not publicly available due to protection of participants privacy and ethical restrictions.
